# Inhibition of IL-10 Production by Maternal Antibodies against Group B Streptococcus GAPDH Confers Immunity to Offspring by Favoring Neutrophil Recruitment

**DOI:** 10.1371/journal.ppat.1002363

**Published:** 2011-11-17

**Authors:** Pedro Madureira, Elva Bonifácio Andrade, Bernardo Gama, Liliana Oliveira, Susana Moreira, Adília Ribeiro, Margarida Correia-Neves, Patrick Trieu-Cuot, Manuel Vilanova, Paula Ferreira

**Affiliations:** 1 ICBAS – Instituto de Ciências Biomédicas de Abel Salazar, Universidade do Porto, Porto, Portugal; 2 IBMC – Instituto de Biologia Molecular e Celular, Porto, Portugal; 3 IBB, Institute for Biotechnology and Bioengineering, Centre of Biological Engineering, University of Minho, Braga, Portugal; 4 Life and Health Sciences Research Institute (ICVS), School of Health Sciences, University of Minho, Braga, Portugal; 5 ICVS/3B's, PT Government Associate Laboratory, Braga/Guimarães, Portugal; 6 Institut Pasteur, Unité de Biologie des Bactéries Pathogènes à Gram-Positif, CNRS URA 2172, Paris, France; Children's Hospital Boston, United States of America

## Abstract

Group B Streptococcus (GBS) is the leading cause of neonatal pneumonia, septicemia, and meningitis. We have previously shown that in adult mice GBS glycolytic enzyme glyceraldehyde-3-phosphate dehydrogenase (GAPDH) is an extracellular virulence factor that induces production of the immunosuppressive cytokine interleukin-10 (IL-10) by the host early upon bacterial infection. Here, we investigate whether immunity to neonatal GBS infection could be achieved through maternal vaccination against bacterial GAPDH. Female BALB/c mice were immunized with rGAPDH and the progeny was infected with a lethal inoculum of GBS strains. Neonatal mice born from mothers immunized with rGAPDH were protected against infection with GBS strains, including the ST-17 highly virulent clone. A similar protective effect was observed in newborns passively immunized with anti-rGAPDH IgG antibodies, or F(ab')_2_ fragments, indicating that protection achieved with rGAPDH vaccination is independent of opsonophagocytic killing of bacteria. Protection against lethal GBS infection through rGAPDH maternal vaccination was due to neutralization of IL-10 production soon after infection. Consequently, IL-10 deficient (IL-10^−/−^) mice pups were as resistant to GBS infection as pups born from vaccinated mothers. We observed that protection was correlated with increased neutrophil trafficking to infected organs. Thus, anti-rGAPDH or anti-IL-10R treatment of mice pups before GBS infection resulted in increased neutrophil numbers and lower bacterial load in infected organs, as compared to newborn mice treated with the respective control antibodies. We showed that mothers immunized with rGAPDH produce neutralizing antibodies that are sufficient to decrease IL-10 production and induce neutrophil recruitment into infected tissues in newborn mice. These results uncover a novel mechanism for GBS virulence in a neonatal host that could be neutralized by vaccination or immunotherapy. As GBS GAPDH is a structurally conserved enzyme that is metabolically essential for bacterial growth in media containing glucose as the sole carbon source (i.e., the blood), this protein constitutes a powerful candidate for the development of a human vaccine against this pathogen.

## Author Summary

*Streptococcus agalactiae* (Group B streptococcus, GBS) is the leading infectious cause of morbidity and mortality among neonates. However, there is still no satisfactory explanation of why neonates are so susceptible to GBS infections. Intrapartum antibiotic prophylaxis (IAP) was implemented in many countries but led to the emergence of antibiotic-resistant GBS strains. Therefore, maternal vaccination represents an attractive alternative to IAP. Here, we show that the high susceptibility of newborn mice to GBS infections is associated with their propensity to produce elevated amounts of immunosuppressive cytokine IL-10. We also demonstrate that IL-10 impairs neutrophil recruitment into infected organs thus preventing bacterial clearance. We identified extracellular GAPDH as the GBS factor that induces the high IL-10 production detected early upon neonatal infection. We show that maternal vaccination with recombinant GAPDH confers robust protective immunity against lethal infection with a GBS hyper-virulent strain in mice offspring. This protection can also be obtained either by antibody neutralization of GBS GAPDH or by blocking IL-10 binding to its receptor. As GBS GAPDH is an essential protein for bacterial growth, it is present in all GBS strains and thus constitutes an appropriate target antigen for a global effective vaccine against this pathogen.

## Introduction

*Streptococcus agalactiae*, also named Group B Streptococcus (GBS), is a Gram-positive encapsulated commensal bacterium of the human intestine that colonizes the vagina of up to 30% of healthy women. This bacterium is the leading cause of neonatal pneumonia, septicemia, and meningitis [1], [2], [3], [4]. Neonatal GBS infections are acquired through maternal transmission and may result in early-onset disease (EOD), which occurs within the first week of life, or in late-onset disease (LOD), that occurs after the first week and accounts for most meningitis cases and deaths [3], [5], [6]. Despite early antimicrobial treatment and improvement in neonatal intensive care, up to 10% of neonatal invasive GBS infections are lethal and 25 to 35% of surviving infants with meningitis experience permanent neurological sequelae [3]. Because recommendations for intrapartum antibiotic prophylaxis (IAP) for mothers in labor at risk for GBS infection have been widely implemented in many countries, the incidence of EOD has declined to <1/1,000 births, but the incidence of LOD has slowly increased in the last decade [7]. An unexpected burden of case fatalities among children aged less than 90 days caused by GBS infection was recently reported in different European countries [8], [9], [10]. Moreover, recent reports described the emergence of antibiotic-resistant GBS strains likely caused by the widespread use of IAP [11], [12].

Maternal vaccination is the best alternative to IAP to deal with GBS neonatal infections. Vaccines to prevent GBS disease have been initially developed by coupling capsular polysaccharide (CPS) antigens to immunogenic protein carriers. Glycoconjugate vaccines against nine GBS serotypes have been shown to be immunogenic in animals, but the existence of distinct epitope-specific capsular serotypes has hampered the development of a global GBS vaccine [5], [13]. Moreover, glycoconjugated vaccines directed against the ten known serotypes of GBS would not protect against infections by nontypeable GBS isolates that are increasingly being reported [14], [15], [16], [17].

The sequencing of numerous GBS genomes has accelerated advances in vaccine development and new protein antigens have been revealed using reverse vaccinology [5], [18], [19]. To avoid the selection of mutants that escape immune recognition, the ideal human GBS vaccine should be directed against structurally conserved antigens that are essential for GBS virulence and/or growth, but none of the hitherto described candidate antigens fulfills these requisites.

The causes for the neonatal susceptibility to GBS infections are still poorly understood. Newborn immune system is not completely developed at birth, and undergoes an age-dependent maturation until fully developed. Thus, invasive infections in the first days of life pose serious threats for the newborn due to accentuated deficiencies in both innate and adaptive arms of the immune responses. Cases of early-onset GBS sepsis are usually characterized by an unexpectedly low number of neutrophils in infected tissues [20], [21], [22], [23]. This is commonly explained by the reduced neutrophil chemotaxis and impaired granulocyte maturation observed in neonates [24], [25], [26]. Of interest, high concentration of plasma and cord blood IL-10 in preterm neonates evaluated for sepsis was associated with mortality and is considered as an early indicator of prognosis [27], [28].

We have previously shown that the essential housekeeping enzyme glyceraldehyde-3-phosphate dehydrogenase (GAPDH) also acts as a GBS extracellular virulence factor that induces rapid production of interleukin-10 (IL-10) by the host [29]. Adult C57BL/6 mice (resistant to GBS infection) infected with a GBS mutant strain that over-express GAPDH (oeGAPDH) had increased bacterial colonization compared to mice infected with wild-type (WT) GBS. Increased bacterial burden in oeGAPDH infected C57BL/6 mice was accompanied by elevated serum levels of IL-10. Consequently, acquired susceptibility of C57BL/6 mice to oeGAPDH infection was completely reverted in IL-10-deficient animals [29]. This suggested that an exacerbated production of IL-10 during GBS infection might facilitate pathogen immune evasion. We demonstrate here that maternal immunization with rGAPDH confers protection against GBS infection in neonatal mice by abrogating the early IL-10 production detected upon the bacterial challenge. We also demonstrate that blocking GAPDH-induced early IL-10 production restores the recruitment of neutrophils in infected organs, which is essential for pathogen elimination and host protection against GBS infection.

Since GBS GAPDH is a structurally conserved enzyme that is metabolically essential for bacterial growth in blood, it constitutes an attractive target for the development of a human vaccine.

## Results

### GAPDH, a structurally conserved GBS protein

**Figure 1:**
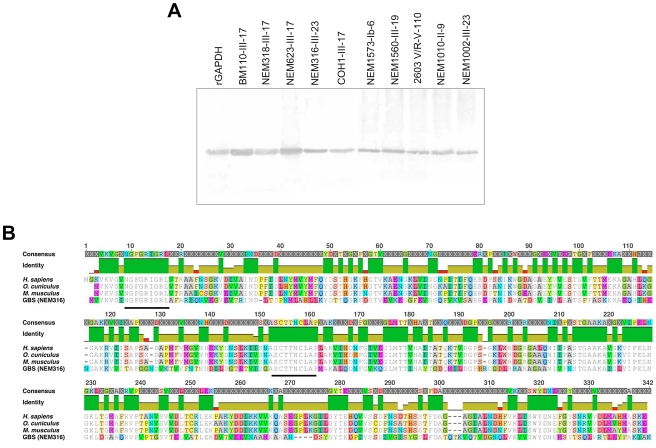
GAPDH is a conserved GBS protein. (A) Extracellular proteins from culture supernatants of different GBS clinical isolates were separated by SDS-page and analyzed by western-blot using anti-rGAPDH IgG obtained from rGAPDH-immunized rabbits. rGAPDH was used as a positive control. The data are representative of four independent experiments. (B) Multiple alignment of the aminoacid sequences of the human, rabbit, mice, and GBS GAPDH. The green vertical bars below the consensus sequences indicate identical aminoacid at the same position in all four sequences. The two heavy black lines delineate the two conserved 10-aminoacid stretches of identity.

**Figure 2:**
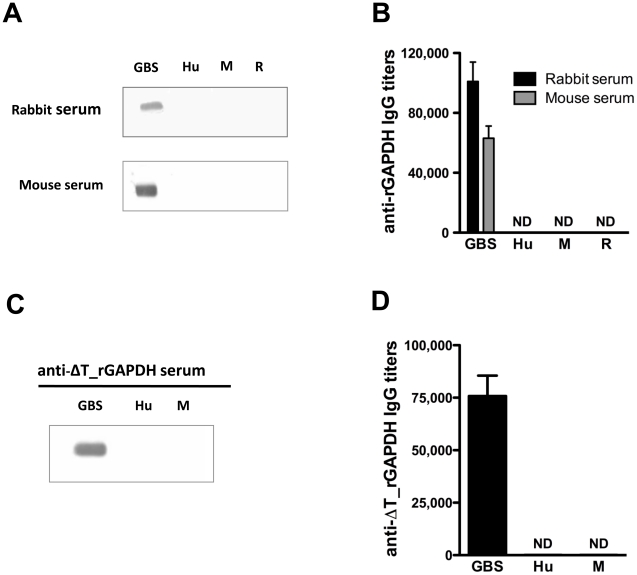
Antibodies against GBS GAPDH do not cross-react with human, rabbit or mouse GAPDH. (A and B) Reactivity of rabbit and mouse anti-rGAPDH IgG against GBS, human (Hu), mouse (M) or rabbit (R) GAPDH were assessed by (A) western-blot or (B) ELISA analysis. (C and D) Reactivity of anti-rGAPDH IgG purified from the sera of mice immunized with heat-denaturated rGAPDH (ΔT_rGAPDH) against GBS, human or mouse GAPDH, were assessed by (C) western-blot or (D) ELISA analysis. ND, not detected.

GAPDH, a key enzyme of the glycolytic pathway, is structurally conserved in all 8 published GBS genomes (identity>99.8%). Anti-rGAPDH IgG antibodies purified from sera of rGAPDH immunized mice or rabbits were thus used to demonstrate the presence of GAPDH in culture supernatants of ten unrelated GBS clinical isolates (Figure 1A) belonging to different serotypes and/or MLSTypes (Table S1). GBS GAPDH displays 44.7, 45.8 and 44.0% amino acid identity with rabbit, mice, and human GAPDH, respectively (Figure 1B). However, western blot and ELISA analysis revealed that rabbits and mice antibodies directed against GBS rGAPDH do not react with human, mouse, or rabbit GAPDH (Figure 2A and 2B). To favor the production of antibodies recognizing linear buried epitopes, mice were immunized with heat-denaturated rGAPDH (ΔT_rGAPDH). Anti-ΔT_rGAPDH antibodies purified from the sera of these animals did not show any cross-reactivity against mouse (self cross-reactivity) or human GAPDH when analyzed by western blot and ELISA (Figure 2C and 2E). These results are consistent with the fact that the longest identical stretches observed between eukaryotic and prokaryotic GADPH sequences are only 10-aminoacid long (Figure 1B).

### Maternal vaccination with rGAPDH protects neonates from GBS infection

To test whether maternal immunization with rGAPDH conferred protection to the offspring against GBS infection, female BALB/c mice were immunized with rGAPDH in alum adjuvant. Control mice were sham-immunized with the adjuvant alone.

**Figure 3:**
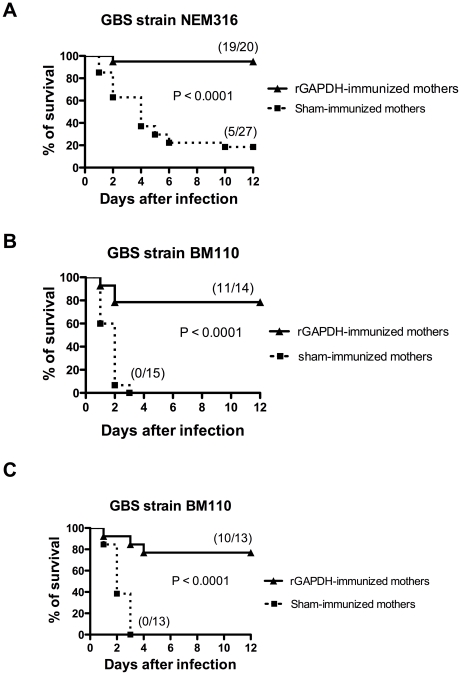
Maternal immunization with rGAPDH protects newborn mice from GBS-induced death. Pups born from sham- or rGAPDH-immunized mothers were infected i.p. 48 h after birth with (A) 5×10^6^ NEM316 CFU or with (B) 10^6^ CFU of the ST-17 hyper virulent strain BM110. (C) Mice pups were infected s.c. 48 h after birth with 2.5×10^4^ CFU of BM110. The results represent data pooled from three independent experiments. In all figures depicting survival experiments, the numbers between parentheses represent the number of animals that survived the different infectious challenges versus the total number of infected animals. Statistical differences (P values) between immunized versus sham-immunized groups are indicated.

Pups born from sham-immunized or rGAPDH-immunized females were infected intraperitoneally (i.p.) 48 h after birth with 5×10^6^ colony-forming units (CFU) of serotype III virulent GBS strain NEM316. All but one mouse born from rGAPDH-immunized mothers survived the infection (95% survival) whereas 22 out of 27 infected pups succumbed to GBS challenge in the control group (18.5% survival) (Figure 3A). Most of the cases of GBS meningitis and LOD are caused by a serotype III hyper virulent clone, defined by multilocus sequence typing as ST-17 [30], [31], [32]. To better assess the effectiveness of maternal vaccination with rGAPDH, pups born from sham- or rGAPDH-immunized progenitors were i.p. infected 48 h after birth with 10^6^ CFU of BM110, a serotype III GBS hyper virulent strain ST-17 (Table S1). All ST-17 GBS challenged neonates born from sham-immunized mothers died whereas mortality rate dropped to 21.4% in neonates born from rGAPDH-immunized mothers (Figure 3B). The protective effect conferred by rGAPDH maternal immunization was also observed in neonate mice infected by the subcutaneous (s.c.) route with 2.5×10^4^ BM110 CFU. As shown in Figure 3C, none of the mice born from sham-immunized mothers survived this infectious challenge whereas only 23% of mice born from rGAPDH-immunized mothers died. Altogether, these results show that maternal vaccination with rGAPDH protected the offspring against GBS infections, including those caused by the hyper virulent strain BM110.

### Passive immunization with anti-rGAPDH F(ab')_2_-fragments protects newborns against GBS ST-17 challenge

**Figure 4:**
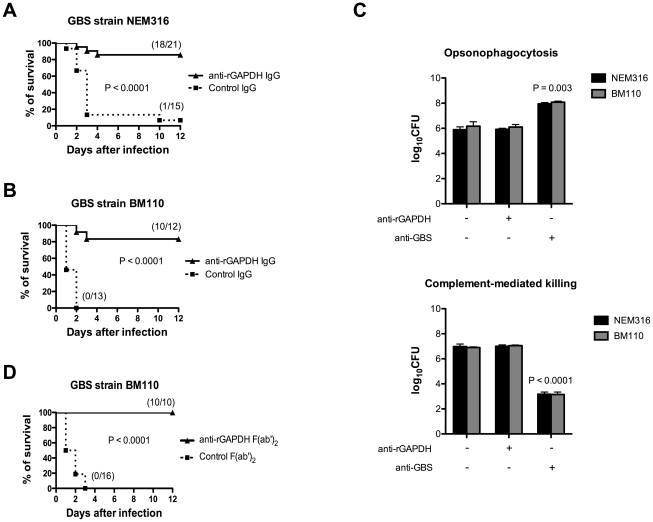
Passive immunization with purified anti-rGAPDH antibodies protects newborn mice from GBS-induced death. anti-rGAPDH IgG or control IgG (80 µg) were injected i.p. into mice pups and 12 h after the immunization they were infected i.p. with (A) 5×10^6^ NEM316 CFU or with (B) 10^6^ CFU of the ST-17 hyper virulent strain BM110. The results represent data pooled from two independent experiments. (C) Upper panel: BMM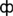 were stimulated *in vitro* with 10^6^ CFU of GBS NEM316 (or BM110) plus 25 µg/mL of anti-rGAPDH IgG's, or 10% of serum containing anti-GBS antibodies, and incubated for 30 min at 37°C in 5% CO_2_. Data represent the mean + SEM. Results are representative of 3 independent experiments. Statistical differences (P values) are indicated. Lower panel: complement-mediated killing of GBS. Mice peripheral blood leukocytes were stimulated with 10^6^ CFU of GBS NEM316 (or BM110) plus 25 µg/mL of anti-rGAPDH IgG's, or 10% of anti-GBS serum, and 5% of rabbit serum was added to the mixture as a source of complement. After 2 h of incubation period at 37°C, GBS CFU were evaluated on agar plates. Data represent the mean + SEM. Results are representative of 2 independent experiments. Statistical differences (P values) are indicated. Similar results were obtained when a higher concentration (100 µg/mL) of anti-rGAPDH IgG's was used. (D) Pups were passively immunized with 80 µg of anti-rGAPDH F(ab')_2_ fragments, or control (Fab')_2_ and infected 12 h later with 10^6^ CFU of the ST-17 hyper virulent strain BM110. The results represent data pooled from two independent experiments.

Pups born from rGAPDH-immunized mothers presented increased serum titers of anti-rGAPDH IgG antibodies when compared with those born from sham-immunized mothers (Figure S1). To evaluate the importance of these maternal antibodies in the newborn protection against GBS infection, neonatal mice were passively immunized with purified anti-rGAPDH IgG antibodies 12 h prior to GBS challenge. The passive antibody transfer conferred protection against infection caused by the virulent NEM316 or hyper virulent BM110 strains (Figure 4A and 4B). Anti-rGAPDH IgG antibodies conferred a similar protection to neonate mice infected by the s.c. route (data not shown). As described by others [33], we observed that GAPDH is present at the cell surface of GBS strains (Figure S2) and, it was therefore conceivable that protection conferred by anti-rGAPDH antibodies could be due to an enhanced opsonophagocytosis-mediated killing of GBS. However, anti-rGAPDH IgG antibodies did not enhanced *in vitro* phagocytosis or complement-mediated killing of GBS BM110 cells (Figure 4C). This indicated that protection conferred by anti-rGAPDH antibodies was not mediated by these mechanisms. Furthermore, complete protection against GBS infection was observed in neonate mice treated with purified anti-rGAPDH F(ab')_2_ fragments 12 h before i.p. infection with BM110 strain. In contrast, all pups that received the same amount of control F(ab')_2_ fragments died within the first 3 days upon the infectious challenge (Figure 4D). Altogether, these results demonstrate that enhanced opsonophagocytic killing or complement activation did not mediate the observed protective effect of anti-rGAPDH antibodies.

### GBS GAPDH induces early IL-10 production in newborn mice

**Figure 5:**
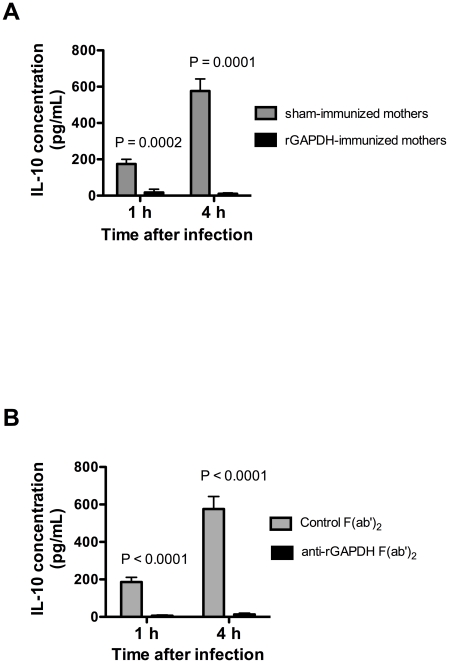
GAPDH neutralization abolishes IL-10 production observed in newborn mice early upon GBS infection. Serum IL-10 concentration in mice pups was measured 1 h and 4 h after i.p. infection with 5×10^6^ NEM316 CFU (A) born from sham- or rGAPDH-immunized dams or (B) passively immunized with anti-rGAPDH F(ab')_2_ fragments or control F(ab')_2_. Data represent the mean + SEM. Results are a representative example out of 3 independent experiments. A minimum of 8 animals per group was used in each experiment. Statistical differences (P values) between immunized versus sham-immunized groups (A) or between pups treated with anti-rGAPDH F(ab')_2_ fragments versus controls (B) are indicated.

We have previously described a rise in IL-10 serum levels in adult mice treated with rGAPDH [29]. As shown in Figure S3, a similar increase in serum IL-10 levels was detected in newborn mice 1 h after i.p. injection of rGAPDH. Inactivation of rGAPDH enzymatic activity did not reduce this effect (Figure S3). This result indicates that IL-10 production induced by GBS GAPDH is independent of the dehydrogenase activity. We have also described that adult mice infected with GBS oeGAPDH mutant strain presented higher serum IL-10 levels than counterparts infected with WT GBS [29]. Thus, we also quantified the levels of serum IL-10 in mice pups at early times after GBS infection. As shown in Figure 5, infection of newborn mice with GBS WT strain NEM316 resulted in a rapid increase of serum IL-10 concentration. Maternal rGAPDH vaccination or treatment with anti-rGAPDH F(ab')_2_ fragments completely abrogated the elevated amount of IL-10 found in the sera of infected pups born from sham-immunized mothers or treated with control F(ab')_2_ (Figure 5A and 5B). Altogether, these results strongly suggest that the elevated IL-10 serum levels detected upon infection were due to GBS GAPDH.

### Protection conferred by anti-rGAPDH antibodies is associated with inhibition of early IL-10 production in GBS-infected pups

**Figure 6:**
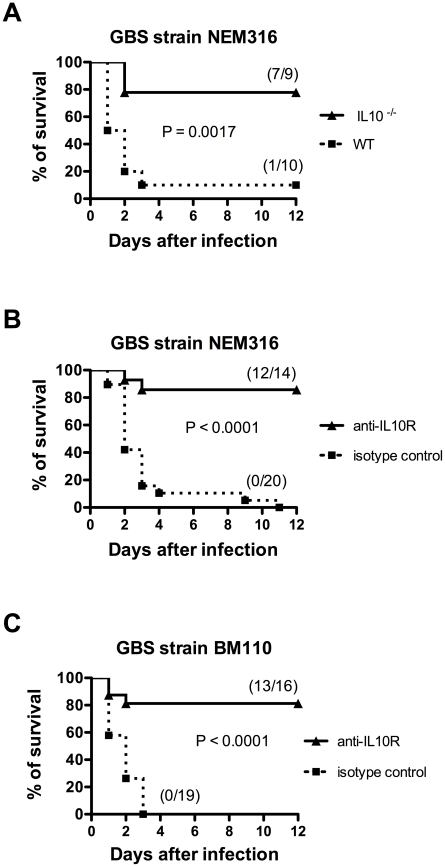
Impairment of IL-10 signaling confers protection to newborn mice against GBS infection. (A) IL10^−/−^ and WT pups were infected i.p. with 5×10^6^ NEM316 CFU (results represent data pooled from two independent experiments). (B) Newborn mice were injected i.p. with anti-IL10R mAb (anti-IL10R) or isotype control IgG (100 µg) and 12 h later were challenged i.p. with 5×10^6^ NEM316 CFU (results represent data pooled from three independent experiments). (C) Newborn mice were injected i.p. with anti-IL10R mAb (anti-IL10R) or isotype control IgG (100 µg) and 12 h later were challenged s.c. with 2.5×10^4^ BM110 CFU (results represent data pooled from four independent experiments). Statistical differences (P values) between IL-10^−/−^ and WT pups (A) or between immunized versus sham-immunized groups (B and C) are indicated.

**Figure 7:**
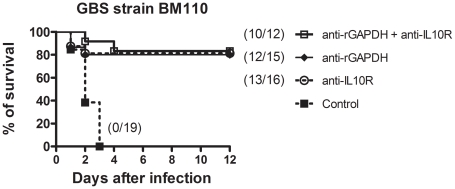
Simultaneous injection of anti-rGAPDH IgG's and anti-IL10R mAb does not increase survival of newborn mice infected with GBS BM110. Newborn mice were treated i.p. with 100 µg of anti-IL10R mAb, anti-rGAPDH IgG's, or simultaneous with anti-IL10R mAb and anti-rGAPDH IgG's (anti-rGAPDH + anti-IL10R) 12 h before s.c. injection of 2.5×10^4^ CFU of GBS BM110. Control pups received 100 µg of control IgG's. P<0.0001 between anti-IL10R-, anti-rGAPDH- and anti-rGAPDH+antiIL-10R-treated pups and controls.

The results presented above indicate that newborn susceptibility to GBS infection is most probably associated with early IL-10 production induced by GAPDH. To confirm this hypothesis, IL-10 deficient (IL-10^−/−^) pups and WT controls were infected with this bacterium. In agreement with our hypothesis, IL-10^−/−^ pups were more resistant (78%) to GBS infection compared to WT controls (10%) (Figure 6A). To demonstrate further the essential role of IL-10 in neonatal susceptibility to GBS, newborn mice were treated with anti-IL-10 receptor (IL-10R) mAb 12 h before NEM316 or BM110 GBS challenge. As expected, most pups treated with anti-IL-10R mAb survived (86% or 82%, respectively) while all control pups died (Figure 6B and 6C). No additional protection was observed when newborn mice were treated simultaneously with anti-IL10R mAb and anti-rGAPDH IgG (Figure 7). Altogether, these results indicate that protection achieved using anti-GAPDH antibodies is due to inhibition of host IL-10 production. Several studies have shown that GBS can survive for prolonged periods within the phagolysosome of macrophages [34], [35], [36], [37]. Interestingly, we observed that the simultaneous addition of anti-rGAPDH IgG's, or anti-IL10R mAb, and GBS cells to bone marrow-derived macrophages (BMMφ) cultures inhibits the bacterial survival (Table 1). This result, combined with those shown in Figure S3, indicates that the inability of the macrophages to kill the intracellular GBS is due to IL-10 production induced by GBS GAPDH.

**Table 1:** Inhibition of GBS intracellular survival in cultured BMM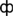.

	Intracellular bacterial count^a^(log_10_CFU ± SEM)			
	NEM316		BM110	
	2 h	24 h	2 h	24 h
**RPMI**	5.21±0.18	6.49±0.88	5.40±0.17	6.67±0.58
**anti-rGAPDH**	4.82±0.08	0.00±0.00**	5.19±0.13	0.00±0.00**
**anti-IL10R**	4.97±0.13	0,66±1.15**	5.14±0.12	0.00±0.00**

a BMM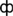 were infected *in vitro* for 2 h with GBS NEM 316 or BM110 at MOI of 10 CFU per macrophage. After three washes in HBSS containing penicillin and streptomycin, infected macrophages were further incubated in RPMI medium containing 10% FCS and antibiotics. At indicated time points, the cells were washed with antibiotic-free HBSS, lysed with saponin, and the CFU estimated by plating serial dilutions of the lysate onto agar plates. ** P<0.0001 compared with RPMI control at the same time point.

### GAPDH blocks neutrophil recruitment in injured organs upon GBS infection

**Figure 8:**
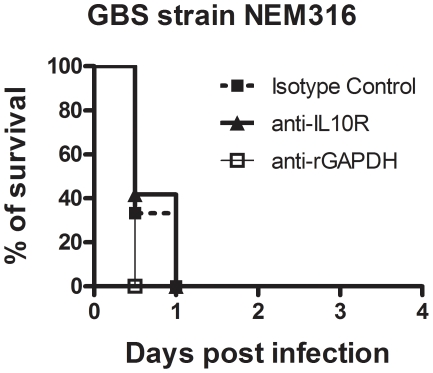
Neutrophils are essential for neonatal protection against GBS infection. Neutropenic mice pups were injected i.p. with 100 µg of anti-rGAPDH antibodies (n = 11), anti-IL-10R mAb (n = 14) or control antibodies (n = 16) 12 h before i.p. infection with 5×10^6^ CFU of NEM316. Results represent data pooled from three independent experiments.

A plausible explanation for the observed protection induced by maternal vaccination with rGAPDH could be that GAPDH-mediated early IL-10 production, elicited upon the GBS challenge in newborns, inhibits the initiation of a host-protective inflammatory response. Neutrophil recruitment is an early event associated with protection against bacterial infection in neonates. Moreover, lack of neutrophil recruitment into infected organs has already been associated with neonatal susceptibility against GBS infections [21], [22], [23], [38]. To confirm that neutrophil recruitment into infected organs is an essential event for newborn protection against GBS infection, neutrophils of newborn mice were depleted by treatment with anti-Ly6G (1A8 clone) monoclonal antibodies (Figure S4). We observed that either blocking IL-10 signaling with neutralizing antibodies or anti-rGAPDH antibody treatment was not sufficient to protect neutropenic pups infected with a lethal inoculum of GBS (Figure 8).

**Figure 9:**
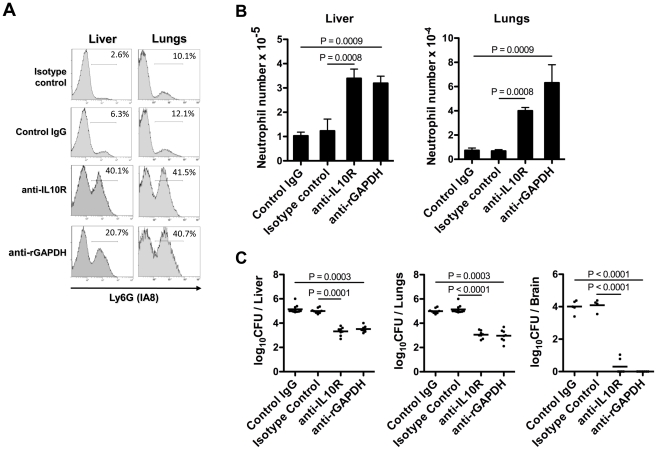
GAPDH-induced IL-10 blocks neutrophil recruitment in injured organs. (A) Flow cytometric analysis of Ly6G expression on total lung or liver leukocyte cells from newborn mice treated i.p. with 100 µg of purified anti-rGAPDH IgG antibodies (anti-rGAPDH), of anti-IL10R mAb (anti-IL10R), or control antibodies (IgG and isotype control) and infected with 5×10^6^ NEM316 CFU 12 h after antibody treatment. Pups were sacrificed 18 h after the bacterial challenge. The percentage of Ly6G^+^ cells is indicated. Results are representative of three independent experiments. (B) Total number of Ly6G^+^ cells (neutrophils) per organ observed in the different used groups. Data are the mean + SEM. Results are representative of 4 independent experiments and a minimal of 5 animals per group was used. Statistical differences (P values) between groups are indicated. (C) GBS CFU in the liver, lungs and brain of infected pups treated with control IgG (n = 11), isotype control IgG (n = 11), anti-IL10R (n = 8), or anti-rGAPDH (n = 8). Statistical differences (P values) between groups are indicated.

In addition, we assessed the numbers and frequency of neutrophils in the liver and lungs of GBS NEM316-infected pups previously treated with anti-rGAPDH IgG or anti-IL10R mAb. The frequency and the total number of neutrophils quantified 18 h after GBS challenge in the analyzed organs of infected pups treated with control IgG (or isotype control) was as low as with the non-infected pups (data not shown). Treatment with either anti-rGAPDH IgG or anti-IL-10R mAb prior to GBS infection significantly increased the neutrophil recruitment in organs (Figure 9A and 9B). Consistently, the organs of pups treated with anti-IL10R mAb or anti-rGAPDH IgG contained significantly less bacteria than those of untreated pups (Figure 9C). These results indicate that an efficient neutrophil recruitment into infected organs is crucial for neonatal protection against GBS infection whereas impaired neutrophil recruitment facilitates GBS colonization. Moreover, no bacterial colonization was detected three weeks after GBS infection in the brain or in any other organ of pups born from rGAPDH-immunized mothers and infected with NEM316 or with the ST-17 hyper-virulent strain BM110 (data not shown). These results indicate that rGAPDH-maternal vaccination is also effective in preventing LOD.

## Discussion

Newborns are highly susceptible to infectious disease and deficiencies of key components of the complement cascade combined with the inability to produce high amounts of antibodies against T-independent antigens greatly impairs their ability to respond to encapsulated bacteria [39]. Absence of previous interactions with environmental microbes also implies that no immunological memory exists against specific antigens, which means that the acquired immune protection of newborns relies mainly on antibodies passively transferred from their mothers [40]. In addition, previous reports indicate that neonatal innate immune cells are less efficient in producing Th1-type inflammatory cytokines, but more competent in producing the immunosuppressive cytokine IL-10 upon Toll-like receptor (TLR) engagement by microbial products [41], [42], [43], [44]. Moreover, newborn mice leukocytes are highly committed to produce increased amounts of IL-10 [44], [45], as also shown in human neonates [41], [43], [46]. As report in this work, high levels of serum IL-10 could be detected in the sera of GBS infected newborn mice early (1 h and 4 h) upon infection. This result is in agreement with a previous report by Cusumano *et al.* [47], showing that elevated levels of plasma IL-10 were detected in newborn mice 24 h and 48 h upon GBS challenge. These authors suggested a host protective role for IL-10 in the outcome of neonatal GBS sepsis as pre-treatment of newborn mice with recombinant IL-10 improved their survival upon a lethal s.c. GBS challenge [47]. Nevertheless, they also showed that a therapeutic administration of this cytokine (24 h after the bacterial challenge) did not improve survival. This would limit its use in human therapies because neonatal GBS infection is usually acquired before or during labor [3], [5], [6].

Our results revealed that blocking IL-10 signaling through anti-IL-10R mAb administration was sufficient to confer protection against a bacterial challenge using either the s.c. or i.p. routes. Thus, they contrastingly indicate that IL-10 has a deleterious effect in the newborn host resistance to GBS infection. The increased resistance of IL-10-deficient neonates to GBS infection reported here constitutes further support for a deleterious effect of IL-10 in host resistance to GBS. As shown here, the GBS GAPDH induced host IL-10 production detected early after bacterial infection.

IL-10 is produced by multiple cell types and inhibits leukocyte activation, pro-inflammatory cytokine production and down-regulates the expression of anti-microbial molecules on activated phagocytes [48], [49], [50], [51]. IL-10 also inhibits production of CC and CXC chemokines by activated monocytes [52], [53], [54]. Since these chemokines are implicated in the recruitment of leukocytes during inflammation, IL-10 production indirectly inhibits leukocyte trafficking to inflamed tissues [50], [55], [56].

IL-10 production was already associated with host susceptibility against different pathogens [24], [45], [57], [58], [59], [60], [61]. We show here that treatment of pups with either anti-IL10R mAb or anti-rGAPDH IgG prior to the GBS challenge increased the neutrophil recruitment in liver and lungs that is triggered upon infection. Neutrophil recruitment is a crucial event in the host effector immune response to GBS [21], [62], [63] and, consequently, lack of neutrophil infiltration in infected sites has been reported in cases of severe early-onset GBS sepsis [21], [22], [23], [38]. Thus, neutralization of GAPDH, and hence blockade of the induced IL-10 production, allowed an effective immune response at an early stage of infection that prevented death of pups. Moreover, pups protected by maternal immunization with rGAPDH presented no GBS CFU in the brain, lungs, and liver 3 weeks upon the infectious challenge. This indicates that protection achieved by this vaccination strategy might prevent LOD.

The recruitment of neutrophils into infected tissues is very important to restrain bacterial replication. Thus, qualitative and quantitative deficiencies in the neutrophils of newborns may explain the observed susceptibility to GBS infections. Indeed, newborn neutrophils have reduced adhesion capabilities due to reduced expression of adhesion molecules [24], [25] and they produced a limited number of microbicidal molecules. Moreover, the number of these cells is also reduced when compared to adults due to insufficiencies on neonatal granulocyte lineage development [26]. As a consequence, the intra-cellular and extracellular killing of pathogens is greatly impaired in neonates [64]. However, our results indicate that, despite these serious functional defects, the neutrophils of neonates can control the GBS infections as long as the inhibitory effect of IL-10 is blocked. Importantly, IL-10 blockade with a specific mAb did not significantly decreased the elevated serum TNF-α levels detected upon GBS infection in neonate mice [47]. This further suggests that impairment of neutrophil recruitment rather than inhibition of pro-inflammatory cytokines could be the prominent effect of IL-10 produced in the course of neonatal GBS infection.

Neonatal sepsis is a pathological condition associated with elevated levels of pro-inflammatory cytokines, including IL-1β, TNF-α, and IL-6. However, it has been previously described that cord blood or plasma IL-10 concentration is significantly increased in neonatal sepsis, constituting an early indicator of prognosis [27], [28]. Of interest, it was also reported that high IL-10 levels are found in children at initial phases of fulminant septic shock [65], [66]. This indicates that early IL-10 production, instead of being a physiological attempt to counterbalance the elevated levels of pro-inflammatory cytokines, could be a predisposing factor for disease. Our results are in accordance with this hypothesis and they provide the first evidence that the lack of neutrophil recruitment in infected organs combined with elevated cord blood IL-10 concentration may account for neonatal susceptibility to GBS infection. Hence, the discovery of GAPDH as an extracellular virulence factor of GBS that induces an early IL-10 production by the infected host could be a significant contribution to our understanding of the pathology of neonatal infections.

GAPDH is a promising candidate for a human GBS vaccine because it is an essential metabolic enzyme that also plays a critical role in virulence. Our results show that maternal vaccination with rGAPDH protects the offspring against GBS lethal infection, including those caused by the hyper virulent ST-17 clone, which is responsible for most cases of neonatal meningitis [31], [32], [38]. As a consequence, maternal rGAPDH vaccination might efficiently protect against both EOD and LOD [5], [67], [68]. We demonstrated that passive immunization of neonates with GAPDH-specific IgG antibodies is sufficient to confer protection against GBS infection. Importantly, rGAPDH maternal vaccination prevents the early production of IL-10 in GBS infected pups and similar protective effect was obtained when GAPDH-specific antibody F(ab')_2_ fragments were used instead of whole IgG. These results indicate that neutralization of GAPDH-mediated IL-10 production, rather than complement activation or bacterial opsonophagocytosis, accounts for the observed protection. The extracellular GAPDH was detected at the bacterial surface and in culture supernatants of GBS isolates, which suggests that neutralization of its biological activity by antibody binding should not be sterically impaired by surface capsular polysaccharides. Recently, Margarit *et al.* showed that pili proteins could be used as a human vaccine to prevent GBS infections but, due to sequence variability, a combination of 3 antigens was required to confer protection against 94% of contemporary GBS strains [19]. It is likely that under selective pressure this vaccine will select GBS variants expressing new pili antigens, as shown for *Neisseria gonorrhoeae* [69], [70], [71], [72], [73]. In contrast, since it is an essential and highly conserved metabolic enzyme, GAPDH is unlikely to accumulate rapidly escape mutations or rearrangements under such a selective immune pressure.

Taken together, our results demonstrate that extracellular GAPDH confers a selective advantage to GBS for survival in the infected host. In particular, GBS GAPDH acts on the host immune system to elicit IL-10 production thereby favoring bacterial colonization and survival. As we demonstrated that GBS GAPDH was still able to induce host IL-10 production upon exposure to an oxidative agent, this mechanism may still operate within the highly oxidative environment resulting from the host inflammatory response. Our data highlight the critical role played by this immunosuppressive cytokine in determining susceptibility to GBS infection at an early time after birth. Our results also show that GBS-associated pathology can be counteracted either by rGAPDH vaccination or IL-10 neutralization. In the future, it will be essential to explore the use of either strategy to induce protection towards other human neonatal pathogens.

## Materials and Methods

### Bacterial strains

Relevant characteristics of the GBS strains used in this study are summarized in Table S1. *Escherichia coli* BL21 (DE3) strain (Novagen) and the pET28a plasmid (Novagen) were used for production of recombinant GAPDH (rGAPDH) as described previously [29]. GBS was grown in Todd-Hewitt broth or agar (Difco Laboratories) containing 0.001 mg/mL of colistin sulphate and 0.5 µg/mL of oxalinic acid (Streptococcus Selective Supplement, Oxoid) and *E. coli* was cultured on Luria-Bertani medium. Bacteria were grown at 37°C.

### Animals

Male and female BALB/c mice (6-8 weeks old) were purchased from Charles River. IL-10-deficient BALB/c (IL-10^−/−^) mice were kindly provided by Dr. A. O'Garra (National Institute for Medical Research, London, U.K.). New Zealand White rabbits were purchased from Charles River. Animals were kept at the animal facilities of the Institute Abel Salazar during the time of the experiments.

### Ethics statement

This study was carried out in strict accordance with the recommendations of the European Convention for the Protection of Vertebrate Animals used for Experimental and Other Scientific Purposes (ETS 123) and 86/609/EEC Directive and Portuguese rules (DL 129/92). The animal experimental protocol was approved by the competent national authority Direcção Geral de Veterinária (DGV) (Protocol Permit Number: 0420/000/000/2008). All animal experiments were planned in order to minimize mice suffering.

### Preparation of active and inactive recombinant GAPDH

Recombinant GAPDH (rGAPDH) was purified as described in detail previously [29]. Enzymatically inactive rGAPDH (inact-rGAPDH) was obtained by pretreatment of the enzyme with 500 µM H_2_O_2_. The lack of enzymatic activity upon inactivation was confirmed using a previously described enzymatic assay for GAPDH [29].

### Maternal immunizations with rGAPDH

Recombinant GAPDH was used for maternal immunization assays. Female mice were injected intraperitoneally (i.p.) twice, with a 3-week intervening period, with 200 µL of a preparation containing 25 µg of rGAPDH in a 1:20 PBS/alum suspension (Aluminium hydroxide Gel; a kind gift of Dr Erik Lindblad, Biosector, Frederickssund, Denmark). The sham-immunized control animals received 200 µL of a 1:20 PBS/alum suspension. Immediately after the second injection, female and male mice were paired. Females were monitored closely during gestation and the day of delivery was recorded. Serum anti-rGAPDH antibody titers were determined by ELISA as previously described [29].

### Antibody treatments

Antibody treatments were performed in newborn BALB/c mice 12 h prior to GBS infection. For passive immunizations, pups were injected i.p. with 100 µg of anti-rGAPDH IgG antibodies or anti-rGAPDH F(ab')_2_ fragments. Control animals received the same amount of control IgG's or F(ab')_2_ fragments issued from control IgG's. For IL-10 signaling blocking, 100 µg of anti-IL10R antibodies (1B1.3a, Schering-Plough Corporation) were administered i.p. and control animals received the same amount of matched isotype control antibody.

### Challenging infections of newborn mice

Newborn mice were infected i.p. with 5×10^6^ cells of GBS NEM316 or 10^6^ cells of GBS BM110 (ST-17), 48 h after birth in a maximum volume of 40 µL. Subcutaneous (s.c.) infections were performed 48 h with 2.5×10^4^ cells of GBS BM110 after birth in a total volume of 20 µL. Survival curves were determined in a 12-day experiment period and newborns were kept with their mothers during the entire time of the experiment. The liver, lungs, and brain of infected pups were aseptically removed at indicated time points and homogenized in PBS and serial dilutions of homogenized organs were plated on Todd-Hewitt agar to enumerate bacterial CFU.

### Purification of anti-rGAPDH IgG antibodies

Adult mice or rabbit were immunized twice with 25 µg of rGAPDH in a PBS/alum suspension as described above and sera were collected 10 days after the second immunization. Pooled serum samples were applied to a Protein G HP affinity column (HiTrap, GE Healthcare Bio-Sciences AB) and purified IgG antibodies were then passed through an affinity column with immobilized rGAPDH (Hi-trap NHS-activated HP, GE Healthcare Bio-Sciences AB). Control IgGs were obtained from sera of mice or rabbits sham-immunized with a PBS/alum suspension and purified on a Protein G HP affinity column. Purified IgG antibody fractions were further equilibrated in PBS and stored at -80°C in frozen aliquots.

### Preparation of anti-GBS serum

GBS-specific antiserum was obtained from mice immunized i.p. twice (with a 3-week interval) with isopropanol-fixed 10^5^ GBS cells plus alum (total volume). Serum from immunized animals (Anti-GBS serum) was obtained from retro-orbital bleeding 10 days after the second immunization.

### Preparation of F(ab')_2_ fragments

F(ab')_2_ fragments from anti-rGAPDH or control IgGs were obtained using IgG1 F(ab) and F(ab')_2_ Preparation Kit (Pierce) used according to manufacturer's instructions.

### Opsonophagocytic assays

Bone marrow-derived macrophages (BMM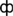) purified as described previously [74] were plated in 96-well plates (10^5^ BMM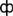/ well) and stimulated for 30 min at 37°C 5%CO_2_ with 10^6^ CFU of GBS BM110 (or NEM316) in the medium alone (RPMI), the medium containing 25 µg/mL of anti-rGAPDH IgG's, or medium with 10% of serum containing anti-GBS IgG antibodies. After this incubation period, the plates were washed three times with HBSS to remove extracellular bacteria. To enumerate intracellular GBS CFU, 10% saponin (1∶100 dilution) was added to wells and serial dilutions of supernatant were plated onto agar plates.

### Complement-mediated killing assay

Blood from adult mice was collected in heparinated tubes and diluted 1:1 in HBSS. 10^6^ GBS NEM316 (or BM110) CFU with 25 µg/mL of anti-rGAPDH IgG's or 10% of serum containing anti-GBS IgG antibodies were then added. Rabbit serum (5%) was added to the mixture as a source of complement. After 2 h of incubation at 37°C, serial dilutions of the mixture were plated onto agar plates to evaluate complement-mediated GBS killing.

### Intracellular survival of GBS in macrophages

The BMM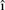, obtained as described previously [74], were infected with GBS strains NEM316 and BM110 at a macrophage:bacteria ratio of 1:10 in RPMI containing 10% FCS. Microplates were incubated for 2 h at 37°C in 5% CO_2_ for GBS phagocytosis. After this period, culture supernatants of infected macrophages were removed by aspiration and cells were washed three times (10 min for each wash) with HBSS containing penicillin (100 IU/mL) and streptomycin (50 µg/mL) to kill extracellular bacteria. Infected macrophages were further incubated in RPMI medium containing 10% FCS and the same concentrations of antibiotics. To quantify intracellular GBS, the supernatants containing antibiotics were removed, the cells were washed with antibiotic-free HBSS, lysed with saponin (0,1% final concentration), and the CFU were estimated by plating serial dilutions of the lysate onto agar plates.

### Neutrophil recruitment

Neutrophil recruitment in liver and lungs of infected pups was evaluated by flow cytometry analysis. Briefly, 18 h after GBS infection, the organs were collected, gently homogenized in HBSS (Sigma), and passed through glass wool to remove cellular aggregates. PerCP/Cy5.5 anti-mouse Ly-6G antibody (clone 1A8; Biolegend) was used for neutrophil detection. Cells were analyzed by an Epics XL cytometer (Beckman Coulter).

### Neutrophil depletion

Newborn mice were depleted of neutrophils by treatment with anti-Ly6G antibodies (clone 1A8, Biolegend). Antibody treatment was performed twice, 12 h before and immediately after GBS challenge. Each pup was injected with a total of 80 µg of anti-Ly-6G antibodies.

### Interleukin-10 quantification

IL-10 was quantitated in the serum of newborn mice with an ELISA kit (eBioscience) used according to the manufacturer's instruction.

### Detection of GAPDH

The presence of GAPDH in the culture supernatants of GBS strains was visualized by Western-blot analysis. Extracellular proteins were isolated as described previously [29]. The reactivity of purified anti-rGAPDH IgG antibodies obtained from the serum of rGAPDH immunized mice or rabbits against self or human GAPDH, was determined by Western-blot analysis or ELISA. Human, rabbit, and mouse GAPDH were purified from human erythrocytes, rabbit erythrocytes or mouse muscle as previously described [75], [76].

### Statistical analysis

Student's T test was used to analyze the differences between groups. Survival studies were analyzed with the log-rank test. A P value<0.05 was considered statistically significant.

## Supporting Information

Figure S1 **Increased anti-rGAPDH IgG serum titers in mice pups born from rGAPDH-vaccinated mothers.** Newborn mice from rGAPDH-immunized mothers present higher serum anti-rGAPDH IgG antibody titers than controls born from sham-immunized mothers. Anti-rGAPDH antibody titers were determined by ELISA. Results are pooled data from three independent experiments (n = 13 or 17 for pups born from sham-immunized or rGAPDH-immunized mothers, respectively).
(TIF)

Figure S2 **GAPDH is present at GBS cell surface.** Fluorescence microscopy analysis of GBS cells using anti-rGAPDH polyclonal antibodies purified from rGAPDH-immunized rabbits and revealed with FITC-conjugated anti-rabbit IgG (Green). Bacterial DNA was stained with DAPI (blue). GBS cells incubated with (A) secondary antibody only, (B) with anti-rGAPDH plus secondary antibody, or (C) with anti-rGAPDH plus rGAPDH to inhibit antibody binding to surface-localized antigen.
(TIF)

Figure S3 **Active or enzymatically inactive rGAPDH induces IL-10 production.** IL-10 concentration in the sera of newborn mice 1 h after i.p. injection with 50 µg of rGAPDH or rGAPDH pre-treated with 500 µM H_2_O_2_ (inact_rGAPDH). Control mice were injected with PBS. Results are pooled data from two independent experiments (n = 9 for controls, 8 for rGAPDH and 7 for pups treated with inact_rGAPDH). Statistical differences (P values) between groups are indicated.
(TIF)

Figure S4 **Treatment of newborn mice with anti-Ly6G antibodies induces neutropenia.** Pups were treated i.p. 36 h and 48 h after birth with 40 µg of anti-Ly6G (clone 1A8) mAb or with the same amount of an isotype matched control antibody. The frequency of blood neutrophils was determined 4 h after the last injection by FACS analysis using anti-Gr-1 mAb (clone RB6-8C5).
(TIF)

Table S1 **Phenotypic and genotypic characteristics of the GBS human isolates used in this study.**
(DOC)
