# Supplementary figures and images for: Inhibition of IL-10 Production by Maternal Antibodies against Group B Streptococcus GAPDH Confers Immunity to Offspring by Favoring Neutrophil Recruitment
Source: PLoS Pathog. 2011 Nov 17;7(11):e1002363. doi: 10.1371/journal.ppat.1002363 (PMC3219712; doi:10.1371/journal.ppat.1002363)

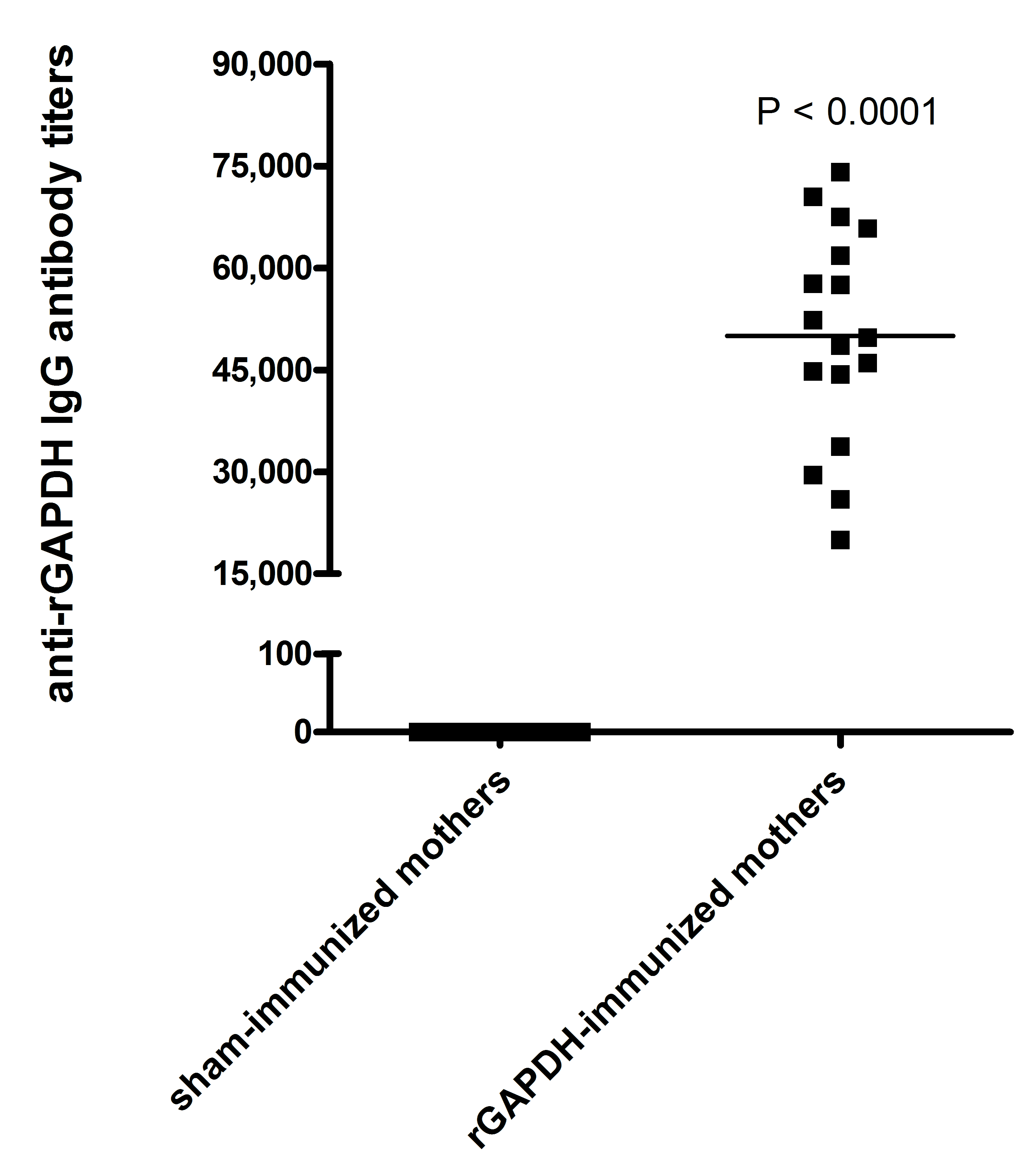

Supplement: Figure S1 — Increased anti-rGAPDH IgG serum titers in mice pups born from rGAPDH-vaccinated mothers. Newborn mice from rGAPDH-immunized mothers present higher serum anti-rGAPDH IgG antibody titers than controls born from sham-immunized mothers. Anti-rGAPDH antibody titers were determined by ELISA. Results are pooled data from three independent experiments (n = 13 or 17 for pups born from sham-immunized or rGAPDH-immunized mothers, respectively). (TIF) [file ppat.1002363.s001.tif]

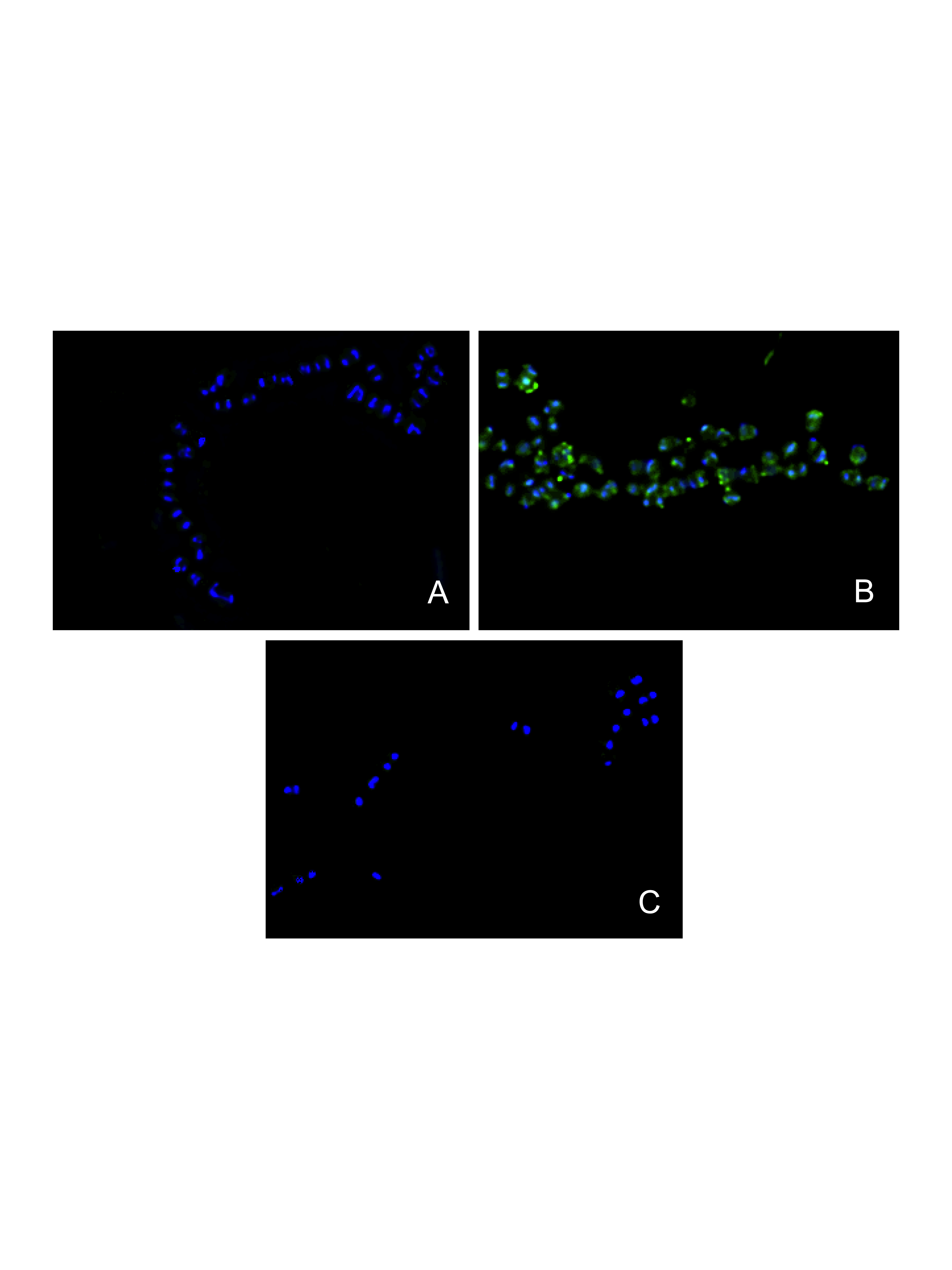

Supplement: Figure S2 — GAPDH is present at GBS cell surface. Fluorescence microscopy analysis of GBS cells using anti-rGAPDH polyclonal antibodies purified from rGAPDH-immunized rabbits and revealed with FITC-conjugated anti-rabbit IgG (Green). Bacterial DNA was stained with DAPI (blue). GBS cells incubated with (A) secondary antibody only, (B) with anti-rGAPDH plus secondary antibody, or (C) with anti-rGAPDH plus rGAPDH to inhibit antibody binding to surface-localized antigen. (TIF) [file ppat.1002363.s002.tif]

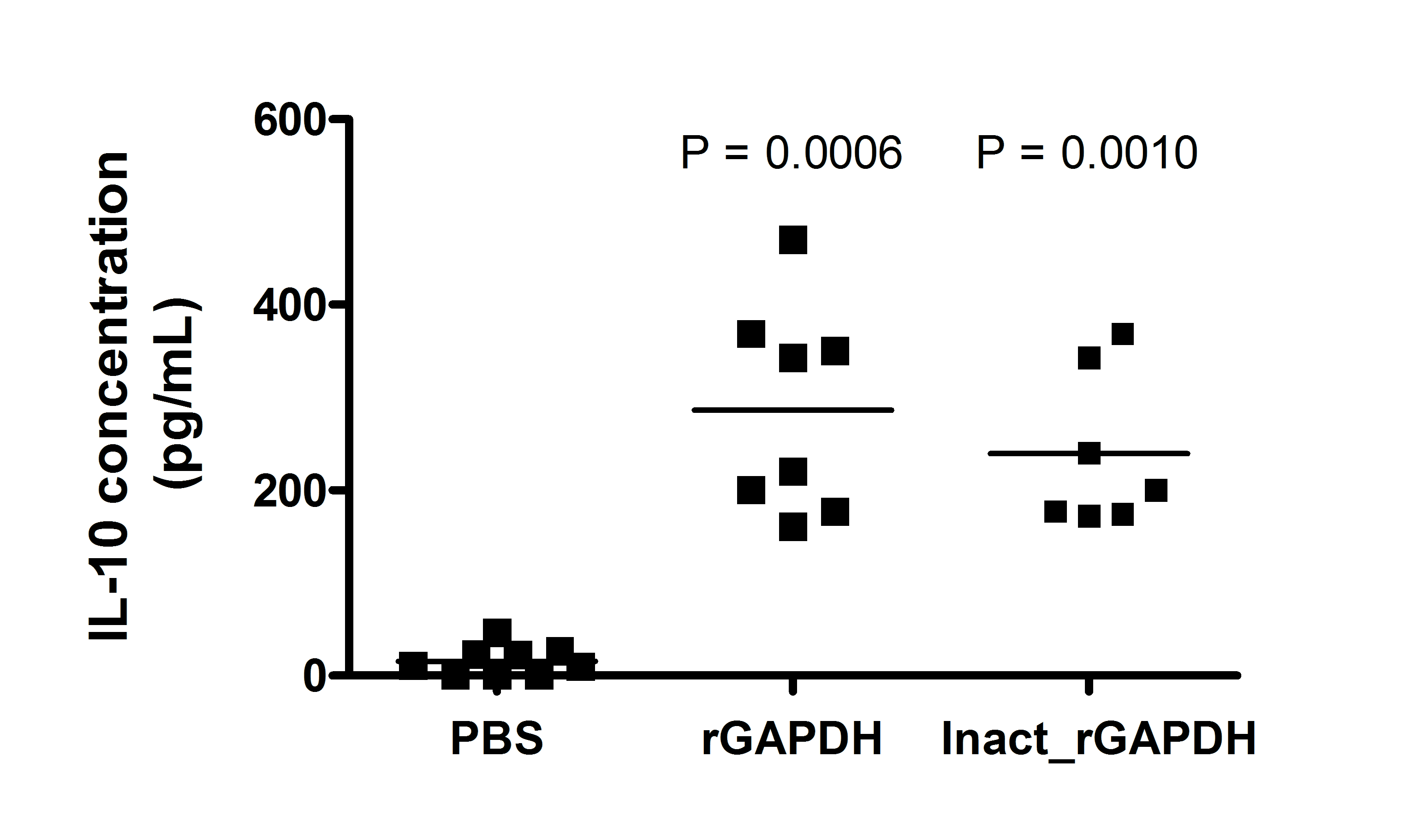

Supplement: Figure S3 — Active or enzymatically inactive rGAPDH induces IL-10 production. IL-10 concentration in the sera of newborn mice 1 h after i.p. injection with 50 µg of rGAPDH or rGAPDH pre-treated with 500 µM H2O2 (inact_rGAPDH). Control mice were injected with PBS. Results are pooled data from two independent experiments (n = 9 for controls, 8 for rGAPDH and 7 for pups treated with inact_rGAPDH). Statistical differences (P values) between groups are indicated. (TIF) [file ppat.1002363.s003.tif]

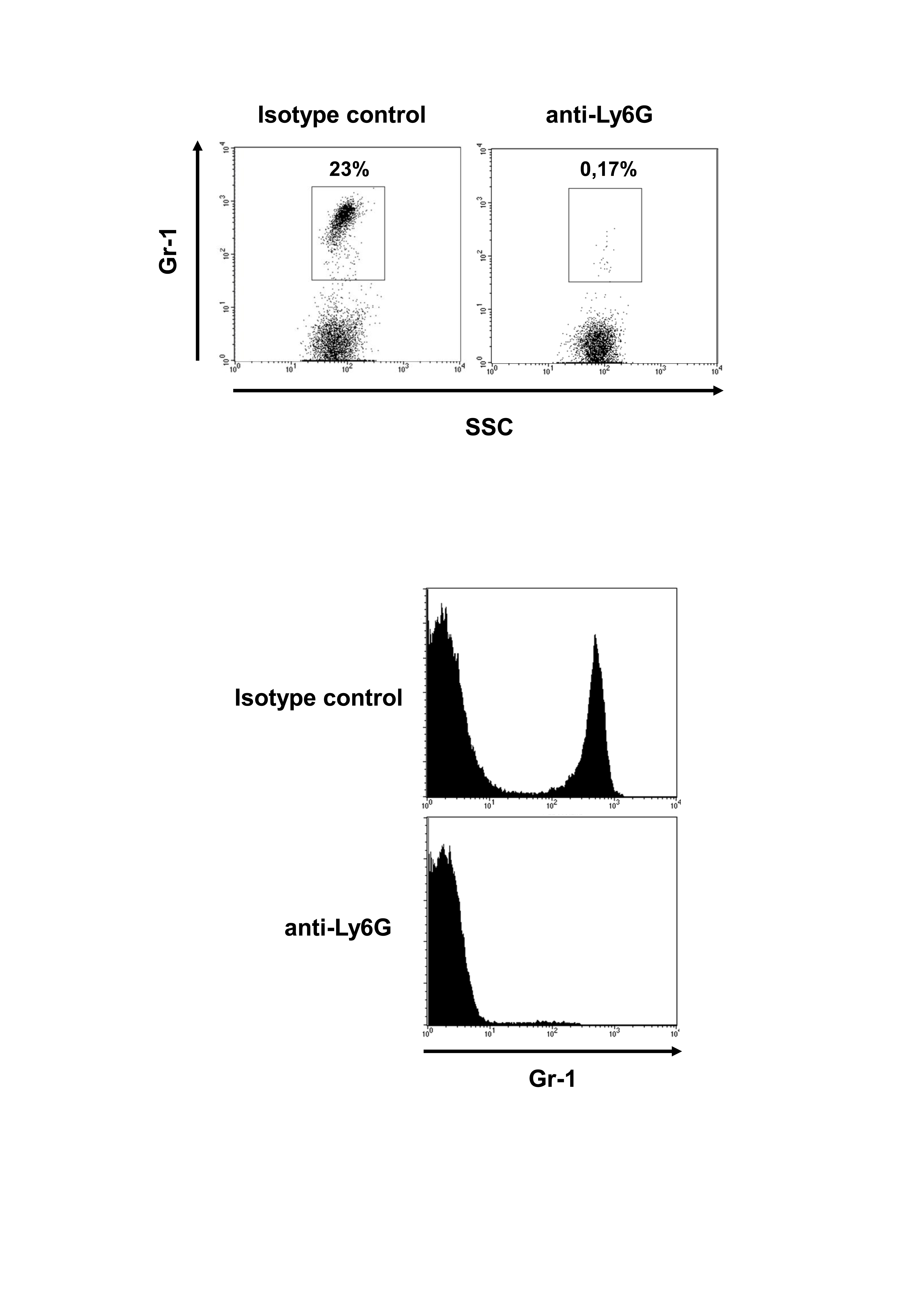

Supplement: Figure S4 — Treatment of newborn mice with anti-Ly6G antibodies induces neutropenia. Pups were treated i.p. 36 h and 48 h after birth with 40 µg of anti-Ly6G (clone 1A8) mAb or with the same amount of an isotype matched control antibody. The frequency of blood neutrophils was determined 4 h after the last injection by FACS analysis using anti-Gr-1 mAb (clone RB6-8C5). (TIF) [file ppat.1002363.s004.tif]
