# Supplementary material for: Inhibition of IL-10 Production by Maternal Antibodies against Group B Streptococcus GAPDH Confers Immunity to Offspring by Favoring Neutrophil Recruitment
Source: PLoS Pathog. 2011 Nov 17;7(11):e1002363. doi: 10.1371/journal.ppat.1002363 (PMC3219712; doi:10.1371/journal.ppat.1002363)
Supplement: Table S1 — Phenotypic and genotypic characteristics of the GBS human isolates used in this study. (DOC) [file ppat.1002363.s005.doc]

| **Strain** | **Origin** | **Serotype** | **MLST** | **Reference** |
| --- | --- | --- | --- | --- |
| NEM316 | Blood, neonate | III | ST-23 | Glaser P, et al. (2002). *Mol Microbiol* 45:1499. |
| NEM318 | CSF, LOD | III | ST-17 | Lamy MC, et al. (2006). *Microbes Infect* 8:1714. |
| NEM623 | Blood, adult | III | ST-17 | Lamy MC, et al. (2006). *Microbes Infect* 8:1714. |
| NEM1002 | CSF, EOD | III | ST-23 | Lamy MC, et al. (2006). *Microbes Infect* 8:1714. |
| NEM1010 | CSF, 3-years-old infant | II | ST-9 | Lamy MC, et al. (2006). *Microbes Infect* 8:1714. |
| NEM1560 | CSF, LOD | III | ST-19 | Lamy MC, et al. (2006). *Microbes Infect* 8:1714. |
| NEM1573 | CSF, EOD | Ib | ST-6 | Lamy MC, et al. (2006). *Microbes Infect* 8:1714. |
| BM110 | Blood, neonate | III | ST-17 | Lamy MC, et al. (2006). *Microbes Infect* 8:1714.  Martin TR, et al. (1988). *J Infect Dis* 157:91. |
| COH1 | Blood, neonate | III | ST-17 | Lamy MC, et al. (2006). *Microbes Infect* 8:1714.  Musser JM, et al. (1989). *Proc Natl Acad Sci U S A* 86:4731. |
| 2603 V/R | Invasive disease, adult | V | ST-110 | Lamy MC, et al. (2006). *Microbes Infect* 8:1714.  Tettelin H, et al. (2002). *Proc Nat Acad Sci U S A* 99:12391. |

**Abbreviations:**

CSF - cerebrospinal fluid; LOD - late onset disease; EOD - early onset disease.
